# Supplementary material for: A long-acting interleukin-7, rhIL-7-hyFc, enhances CAR T cell expansion, persistence, and anti-tumor activity
Source: Nat Commun. 2022 Jun 13;13:3296. doi: 10.1038/s41467-022-30860-0 (PMC9192727; doi:10.1038/s41467-022-30860-0)
Supplement: Supplementary file 1 — Supplementary Information [file 41467_2022_30860_MOESM1_ESM.pdf]

A long-acting interleukin-7, rhIL-7-hyFc, enhances CAR T cell expansion, persistence and anti-tumor activity

Kim et al.

Supplementary Information

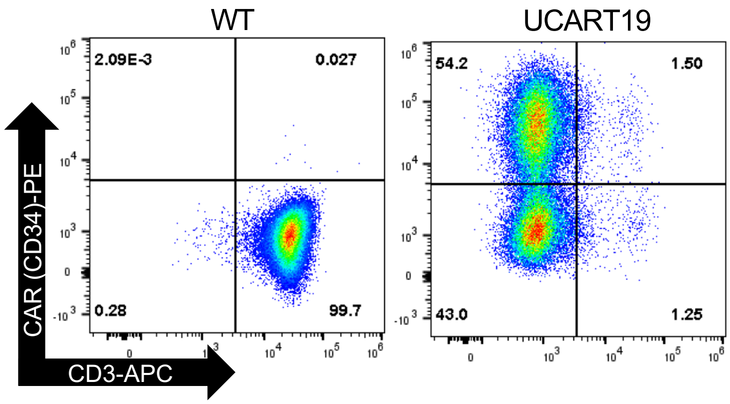

Supplementary Figure 1. Efficiency of TCR deletion and CAR transduction for UCART19. Representative FACS plots of wild-type (WT) T cells and UCART19. CRISPR/Cas9-mediated *TRAC* deletion efficiency was consistently >97%, and transduction efficiency ranged 50-64% (average 58%, n=4 donors).

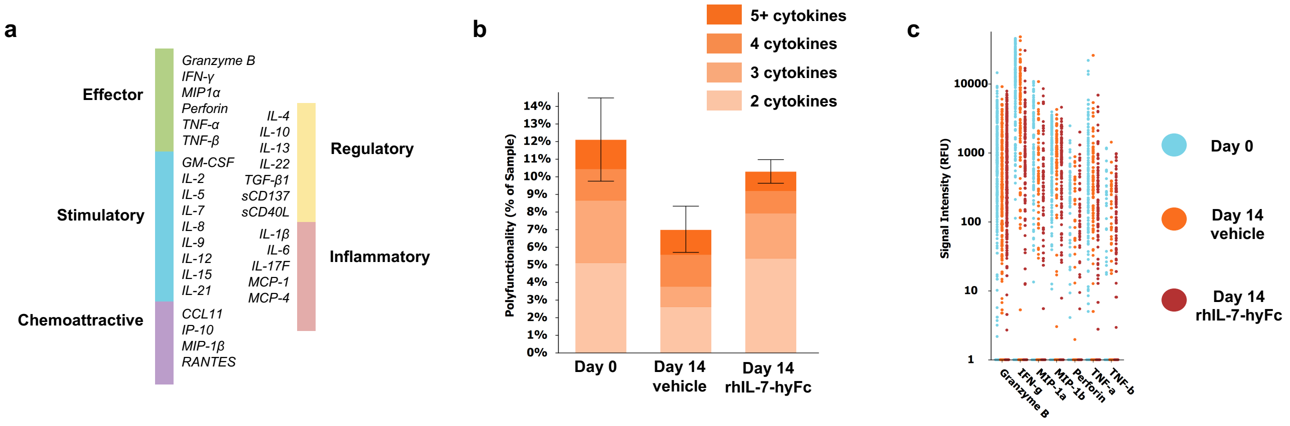

Supplementary Figure 2. Isocode assay shows T cells retain polyfunctionality after rhIL-7-hyFc mediated expansion. UCART19 was incubated with Ramos at an E:T of 1:1 for 16hrs with either vehicle or rhIL-7y-hyFc (100ng/ml) prior to loading purified CAR T cells onto the IsoCode chip for single cell multiplex cytokine analysis. **a** Schema of the different cytokines assessed by the IsoCode assay. **b** Polyfunctionality, as defined by cells cosecreting at least 2 proteins, of UCART19 on Day 0 (UCART19 input cells), Day 14 with vehicle, or Day 14 with rhIL-7-hyFc. Bar graphs represent mean ± standard error of the mean (n=1 donor, 2 technical replicates for Day 0, 3 technical replicates for Day 14 vehicle/rhIL-7-hyFc). **c** Signal strength of key cytokines driving polyfunctionality for each group.

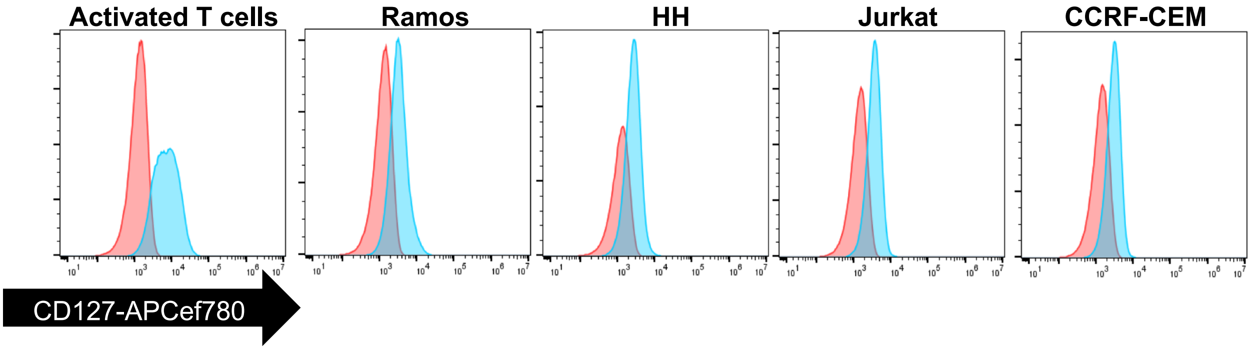

Supplementary Figure 3. Cell line expression of IL7 receptor. Histograms of IL7Rα expression on primary T cells and malignant cell lines, determined by flow cytometry. Blue = stained with anti- CD127 APC-eFluor® 780. Red = Unstained controls.

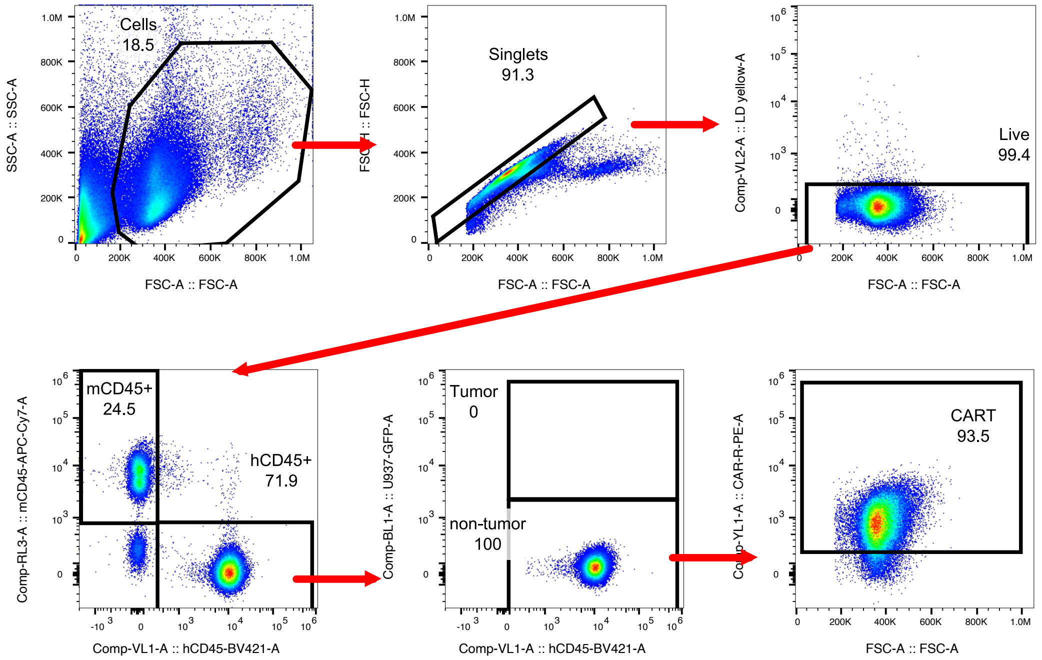

Supplementary Figure 4. Gating strategy to identify UCART populations in NSG mice. NSG mice were injected with either Ramos$^{CBR\text{-}GFP}$ or U937$^{CBR\text{-}GFP}$ on day -4 followed by 1x10^{6} UCART19 or UCART33 on day 0, and treated with rhIL-7-hyFc every two weeks for a total of three doses starting on day +1. Flow cytometry was used to quantify numbers of peripheral blood human CAR T cells (Fig. 2e, f and Fig. 3e). UCART was defined as human CD45+ GFP- CAR+ cells. For UCART19, CAR was stained using a human CD34-PE antibody; for UCART33, CAR was stained using PE-labeled CD33 protein.

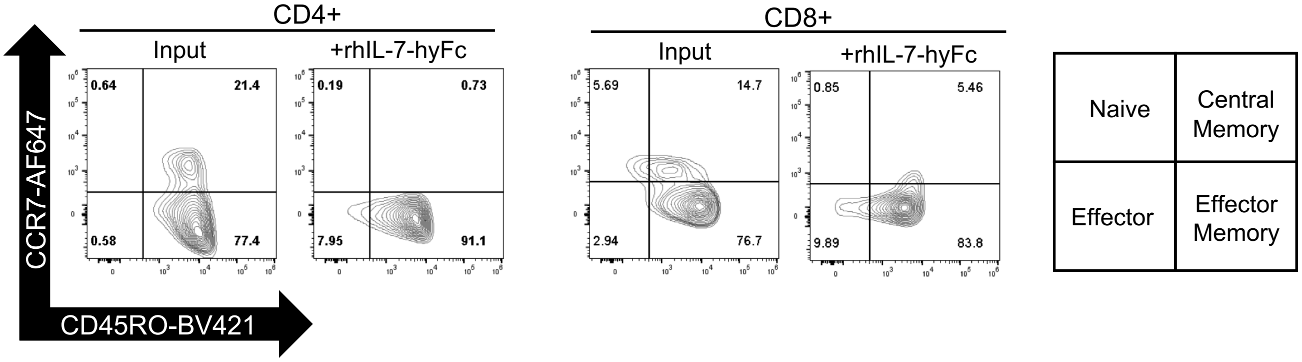

Supplementary Figure 5. In vivo treatment with rhIL-7-hyFc leads to a predominantly effector memory phenotype of UCART19. NSG mice were injected via tail vein with $5x10^5$ Ramos$^{CBR\text{-}GFP}$ on day -4 followed by $1x10^6$ UCART19 on day 0 and treatment with rhIL-7-hyFc on day +1 and day +15. T cell memory subsets were evaluated for CD4+ and CD8+ UCART19 cells from the initial injected product on day 0 (input) and UCART19 derived from rhIL-7-hyFc treated mice on day +28.

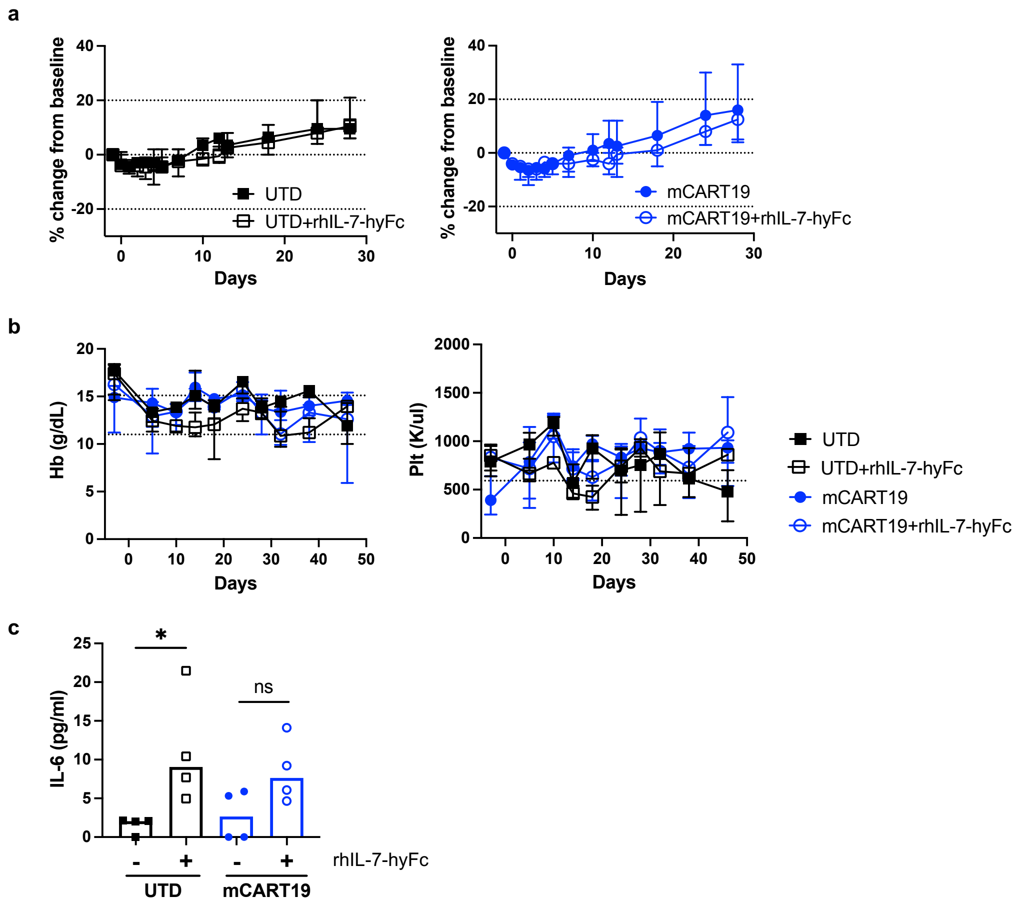

Supplementary Figure 6. rhIL-7-hyFc does not cause any notable toxicities in immunocompetent mice. C57BL/6J mice were injected with Cytoxan (day -1) followed by UTD or mCART19 (day 0), then given serial injections of rhIL-7-hyFc (day +1, +15, +29) as in Figure 4A (n=4 mice/group). **a** Mice were weighed daily from day -1 to day +5, then weighed every 2-4 days until day +28. Data are presented as median ± range. **b** Serial measurements of peripheral blood hemoglobin (Hb) levels (dashed lines indicates upper and lower limit of normal) and platelet (Plt) levels (dashed line indicates lower limit of normal). Data are presented as median ± range. **c** Plasma IL-6 levels measured on day +5. Bar graphs represent median values, with each data point representing one mouse. P-values were determined by two-sided unpaired Student's t-test (UTD: + vs. – rhIL-7-hyFc, p=0.0395, mCART19: + vs. – rhIL-7-hyFc, p=0.0747). Source data are provided as a Source Data file. ns: not significant, *p ≤ 0.05.

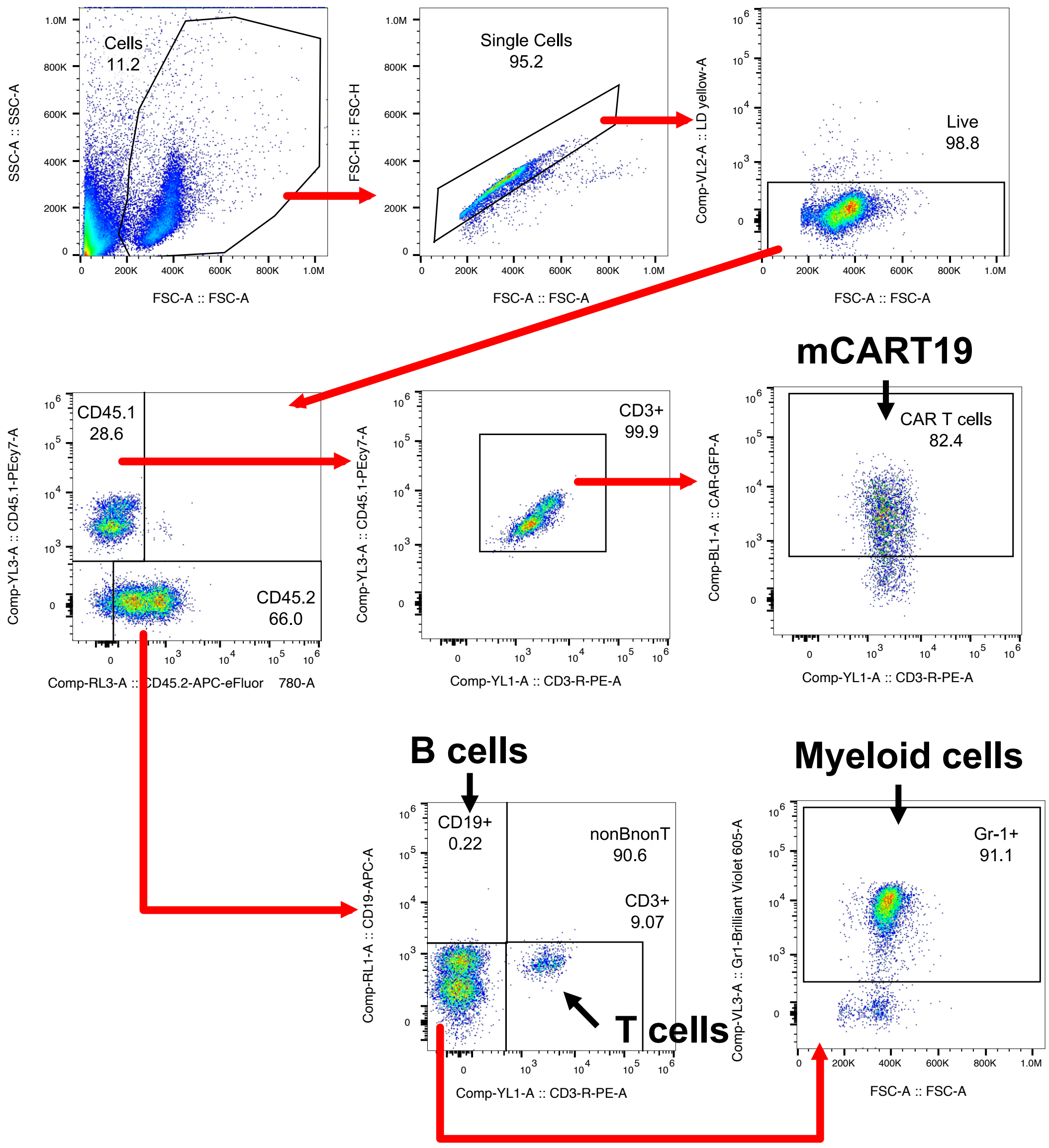

Supplementary Figure 7. Gating strategy to identify peripheral blood subsets in immunocompetent mice. C57BL/6 or Balb/c mice (both CD45.2+) were engrafted with syngeneic tumor cells then treated with congenic CD45.1+ mCART19 followed by rhIL-7-hyFc injections. Flow cytometry was used to quantify numbers of peripheral blood endogenous B cells, T cells and myeloid cells, and mCART19 cells (Fig. 4d, e, f, Fig. 5b, and Fig. 6c, d, e). CAR T cells were identified as CD45.1+CD3+GFP+ cells. Endogenous CD45.2+ cells were classified as B cells (CD19+), T cells (CD3+), and myeloid cells (Gr-1+).

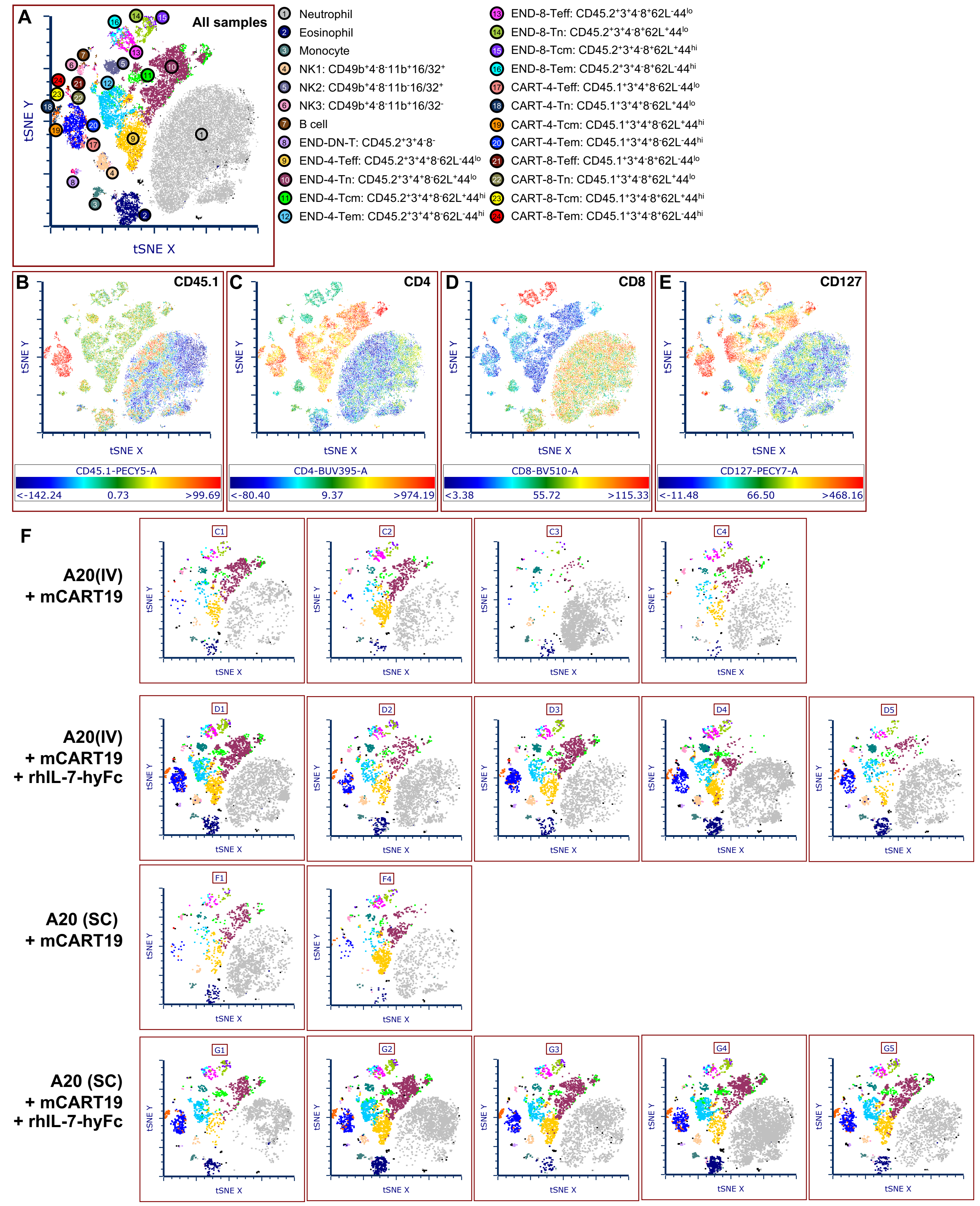

Supplementary Figure 8. Flow cytometry analysis of T cell subsets in Balb/c mice after mCART19 and rhIL-7-hyFc. a t-SNE representation of flow cytometry data from all CD45+ cells from all mice, with relevant subsets marked in the legend. **b-e** Heatmaps of four different parameters included in the t-SNE analysis. Cells colored according to expression of CD45.1 (**b**; mCART19 cells are CD45.1+), CD4 (**c**) CD8 (**d**) and CD127 (IL7Ra; **e**). **f** t-SNE plots shown for each mouse in each group.

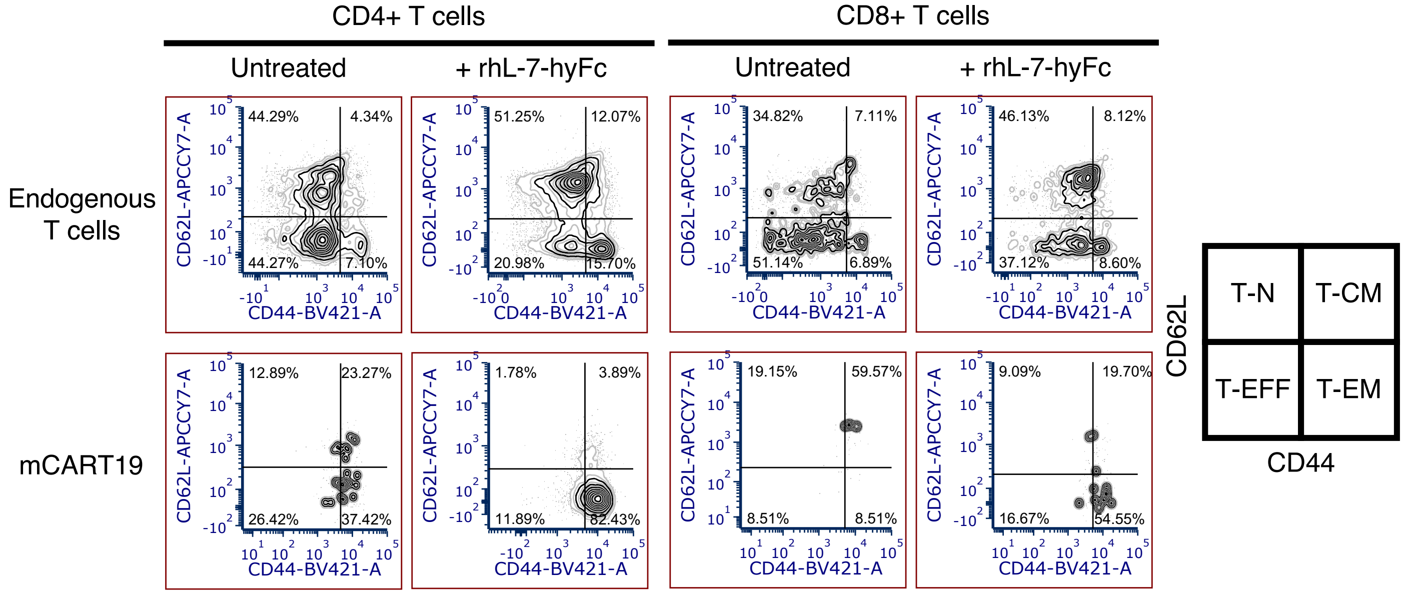

Supplementary Figure 9. In vivo treatment with rhIL-7-hyFc leads to a predominantly effector memory phenotype of mCART19. Balb/c mice were given A20 tumor cells followed by mCART19 cells and serial injections of rhIL-7-hyFc as in Figure 5. Peripheral blood cells T cell subsets were evaluated by flow cytometry on day +57.

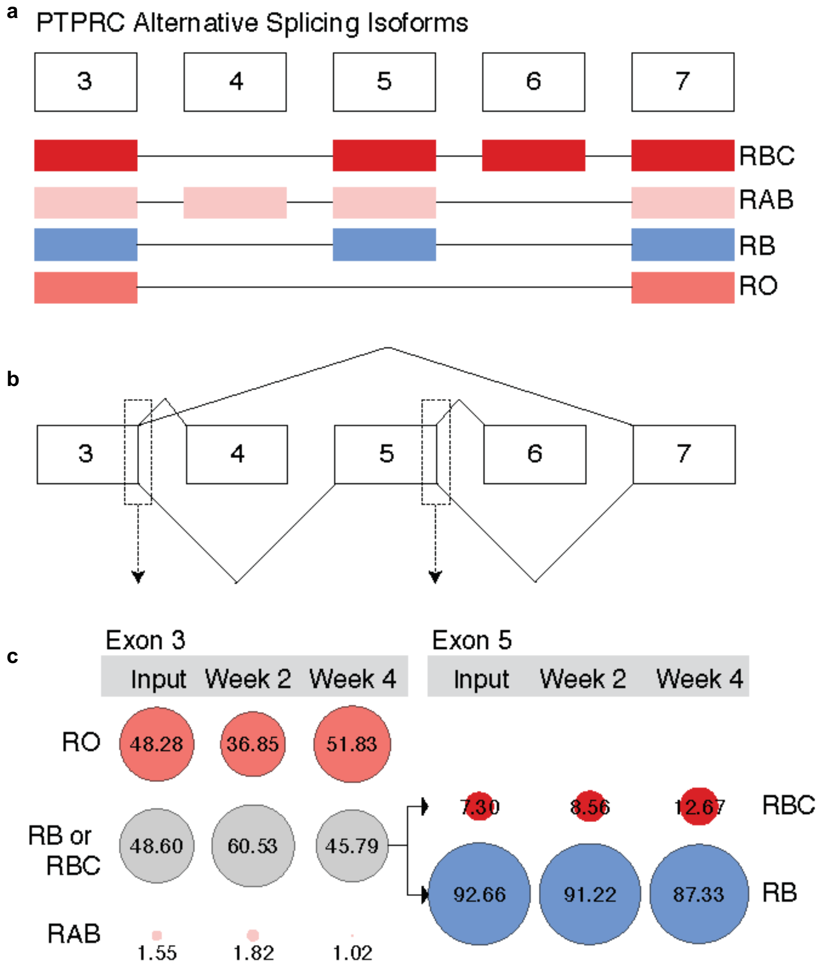

Supplementary Figure 10. Single cell RNA sequencing analysis of CD45 isoform expression. a Alternative splicing cartoon of PTPRC (CD45). Each exon (square) connected by introns (line) has a different combination for each PTPRC isoform (RBC- red, RAB- pink, RB – blue, RO - salmon). **b** Connections between upstream and downstream exons that were analyzed in the exon junction analysis. **c** Proportion of split reads supporting each exon-exon connection (exon junction), indicated by size of the circle and the value is within the circle, broken down by isoform and treatment group
