# Supplementary material for: A long-acting interleukin-7, rhIL-7-hyFc, enhances CAR T cell expansion, persistence, and anti-tumor activity
Source: Nat Commun. 2022 Jun 13;13:3296. doi: 10.1038/s41467-022-30860-0 (PMC9192727; doi:10.1038/s41467-022-30860-0)
Supplement: Supplementary file 3 — Reporting Summary [file 41467_2022_30860_MOESM3_ESM.pdf]

nature portfolio

Corresponding author(s): John F. DiPersio

Last updated by author(s): 2/15/2022

Reporting Summary

Nature Portfolio wishes to improve the reproducibility of the work that we publish. This form provides structure for consistency and transparency in reporting. For further information on Nature Portfolio policies, see our Editorial Policies and the Editorial Policy Checklist.

Statistics

For all statistical analyses, confirm that the following items are present in the figure legend, table legend, main text, or Methods section.

n/a	Confirmed	
☐	☒	The exact sample size (*n*) for each experimental group/condition, given as a discrete number and unit of measurement
☐	☒	A statement on whether measurements were taken from distinct samples or whether the same sample was measured repeatedly
☐	☒	The statistical test(s) used AND whether they are one- or two-sided<br>*Only common tests should be described solely by name; describe more complex techniques in the Methods section.*
☐	☒	A description of all covariates tested
☐	☒	A description of any assumptions or corrections, such as tests of normality and adjustment for multiple comparisons
☐	☒	A full description of the statistical parameters including central tendency (e.g. means) or other basic estimates (e.g. regression coefficient) AND variation (e.g. standard deviation) or associated estimates of uncertainty (e.g. confidence intervals)
☐	☒	For null hypothesis testing, the test statistic (e.g. *F*, *t*, *r*) with confidence intervals, effect sizes, degrees of freedom and *P* value noted<br>*Give P values as exact values whenever suitable.*
☒	☐	For Bayesian analysis, information on the choice of priors and Markov chain Monte Carlo settings
☒	☐	For hierarchical and complex designs, identification of the appropriate level for tests and full reporting of outcomes
☒	☐	Estimates of effect sizes (e.g. Cohen's *d*, Pearson's *r*), indicating how they were calculated

Our web collection on statistics for biologists contains articles on many of the points above.

Software and code

Policy information about availability of computer code

Data collection	Flow cytometry data were collected using pre-installed software on an Attune NxT Flow cytometer (Thermo Fisher Scientific) or a ZE5 (Yeti) cytometer (Bio-Rad). Bioluminescence data was collected using Aura 4.0 In Vivo Imaging Software (Spectral Instruments). Single-cell cytokine profiling was performed using an IsoLight System (IsoPlexis).
Data analysis	Flow cytometry data were analyzed using Flowjo 10.6.1 (TreeStar) or FCS Express v7.08.0018 (De Novo Software). Bioluminescence images were analyzed using Aura 4.0 In Vivo Imaging Software (Spectral Instruments). Polyfunctional profile of single cells was evaluated by IsoSpeak software version 2.7.0.0. Data plots were generated with GraphPad Prism 9.1.0. The statistical analyses were performed using SAS 9.4 (SAS Institutes). scRNA-seq was analyzed using the following software: CellRanger v4.0.0, R v3.6, Python v3.7, Seurat v3.1.2 and v4.0.3.

For manuscripts utilizing custom algorithms or software that are central to the research but not yet described in published literature, software must be made available to editors and reviewers. We strongly encourage code deposition in a community repository (e.g. GitHub). See the Nature Portfolio guidelines for submitting code & software for further information.

Data

Policy information about availability of data

All manuscripts must include a data availability statement. This statement should provide the following information, where applicable:

- Accession codes, unique identifiers, or web links for publicly available datasets
- A description of any restrictions on data availability
- For clinical datasets or third party data, please ensure that the statement adheres to our policy

All data generated from this study are available within the paper and its supplementary information. Source data are provided with this paper. Human and mouse reference genomes (refdata-gex-GRCh38-and-mm10-2020-A) required for CellRanger can be downloaded from 10x Genomics (https://support.10xgenomics.com/

single-cell-gene-expression/software/downloads/latest). The scRNA-seq data generated in this study has been deposited into the NCBI sequence read archive (SRA) database under BioProject accession PRJNA789884 (https://www.ncbi.nlm.nih.gov/bioproject/789884).

Field-specific reporting

Please select the one below that is the best fit for your research. If you are not sure, read the appropriate sections before making your selection.

☒ Life sciences ☐ Behavioural & social sciences ☐ Ecological, evolutionary & environmental sciences

For a reference copy of the document with all sections, see nature.com/documents/nr-reporting-summary-flat.pdf

Life sciences study design

All studies must disclose on these points even when the disclosure is negative.

Sample size	Sample size was not predetermined by statistical methods. For in vitro studies, a minimum of three biological replicates were assayed to allow for statistical evaluation. For in vivo experiments, a minimum of 4 animals per treatment group were evaluated to observe statistically significant differences. The magnitude of effect observed was generally large enough to compensate for small sample sizes.
Data exclusions	No data were excluded from this study.
Replication	The numbers of biological replicates for each experiment is indicated in the figure legends. In vitro experiments in Figure 1a-e were performed independently twice; all other experiments were performed once. Each in vivo experiment was performed once in each model. We elected to replicate the effects of rhIL-7-hyFc between different models, using different CAR constructs (UCART19 and UCART33), different species (human and mouse), and different strains of mice (C57BL/6J and BALB/c), rather than repeat experiments within each model.
Randomization	For in vitro studies, identical cell samples were divided into different treatment groups, and no randomization was required. For in vivo studies, allocation of animals was random to each treatment group, and all mice were matched for age and sex within the same experiment.
Blinding	Data collection and analysis was performed by an independent technician who was blinded to the treatment groups.

Reporting for specific materials, systems and methods

We require information from authors about some types of materials, experimental systems and methods used in many studies. Here, indicate whether each material, system or method listed is relevant to your study. If you are not sure if a list item applies to your research, read the appropriate section before selecting a response.

Materials & experimental systems

n/a	Involved in the study	
☐	☒	Antibodies
☐	☒	Eukaryotic cell lines
☒	☐	Palaeontology and archaeology
☐	☒	Animals and other organisms
☒	☐	Human research participants
☒	☐	Clinical data
☒	☐	Dual use research of concern

Methods

n/a	Involved in the study	
☒	☐	ChIP-seq
☐	☒	Flow cytometry
☒	☐	MRI-based neuroimaging

Antibodies

Antibodies used	CD34-PE (Beckman Coulter, IM1459U, dilution 1:30), LIVE/DEAD fixable yellow (Thermo Fisher Scientific, L34967, dilution 1:500), Annexin V-APC (BD Biosciences, 550474, dilution 1:10), Ki-67-APC (eBioscience, 17-5698-82, dilution 1:10), CD4-FITC (BioLegend, 300506, dilution 1:200), CD8-BV421 (BioLegend, 301036, dilution 1:80). Fluorescently labeled CD33 protein (Sino Biological, 12238-H05H-100, dilution 1:500), CD45RO-BV421 (BioLegend, 304224, dilution 1:100), CD45RA-APC/Cy7 (Biolegend, 304128, dilution 1:100), CCR7-AF647 (BioLegend, 353218, dilution 1:100), CD3-BV786 (BioLegend, 317330, dilution 1:100), CD4-BV650 (BioLegend, 317436, dilution 1:100), CD8-AF700 (BioLegend, 300922, dilution 1:400), CD45.1-PE/Cy7 (eBioscience, 25-0453-82, dilution 1:200), CD45.2-APC/ef780 (eBioscience, 47-0454-82, dilution 1:100), CD19-APC (BioLegend, 115512, dilution 1:400), Gr-1-BV605 (BioLegend, 108440, dilution 1:500), CD3-PE (BioLegend, 100206, dilution 1:100), CD4-BUV395 (BD, 563790, dilution 1:400), LIVE/DEAD fixable blue (Thermo Fisher Scientific, L23105, dilution 1:500), CD11b-BUV661 (BD, 565080, dilution 1:500), B220-BUV737 (BD, 612838, dilution 1:500), CD44-BV421 (BD, 563970, dilution 1:500), CD8-BV510 (BioLegend, 100752, dilution 1:500), CD45.2-BV605 (BioLegend, 109841, dilution 1:500), Ly6G-BV650 (BioLegend, 127641, dilution 1:500), CD16/32-BV711 (BioLegend, 101337, dilution 1:500), CD49b-BV785 (BD, 740895, dilution 1:500), CD19-PerCP/C5.5 (BioLegend, 152405, dilution 1:500), Ly6C-PE-CF594 (BD, 562728, dilution 1:1000), CD45.1-PE/Cy5 (eBioscience, 15-0453-82, dilution 1:1000), CD127-PE/Cy7 (eBioscience, 25-1273-82, dilution 1:1000), CD3-APC (BD, 553066, dilution 1:500), CD62L-APC/Cy7 (BioLegend, 104428, dilution 1:500).
Validation	Antibody validation was performed by the manufacturers (BioLegend, BD Bioseceinces, Beckman Coulter, eBioscience) using primary

Validation	human peripheral blood mononuclear cells (PBMCs), murine splenocytes or antigen-expressing cell lines. The staining profile of each antibody was also confirmed prior to use by titration on normal PBMCs or antigen-expressing tumor cell lines (mixed with antigen-negative control cells) prior to use.

Eukaryotic cell lines

Policy information about cell lines

Cell line source(s)	Ramos (CRL-1596), NALM6 (CRL-3273), U937 (CRL-1593.2), and A20 (TIB-208) cell lines were obtained directly from ATCC. 9523 murine acute promyelocytic leukemia (APL) cells were generated in our lab.
Authentication	Cell lines were authenticated prior to use by flow cytometry to evaluate for expression of the target antigens (CD19, CD33).
Mycoplasma contamination	Cell lines were tested quarterly for mycoplasma. All cell lines used in these studies were negative for mycoplasma contamination.
Commonly misidentified lines (See ICLAC register)	No commonly misidentified cell lines were used in this study.

Animals and other organisms

Policy information about studies involving animals; ARRIVE guidelines recommended for reporting animal research

Laboratory animals	All mice used in these studies were aged 6-12 weeks old males purchased from Jackson Laboratories (Bar Harbor, ME): NOD-SCID-IL2R-/- (NSG) (#005557), C57BL/6J (#000664), BALB/cJ (#000651), B6.SJL- Ptprcaa Pepcb/BoyJ (B6-CD45.1) (#002014), CByJ.SJL(B6)-Ptprcaa/J (Balb/c-CD45.1) (#006584). All animal experiments were performed according to an animal protocol approved by the Institutional Animal Care and Use Committee at Washington University School of Medicine. All experimental mice were co-housed within specific pathogen free facilities at Washington University School of Medicine and maintained on ad libitum water and standard chow (LabDiet 5053; Lab Supply, Fort Worth, TX), with a 12 hour light/dark cycle and a temperature range of 68-74F with 40-60% humidity. Mice were euthanized if they exhibited signs of illness or discomfort (tumor growth >=2cm, weight loss >=20%, hind limb paralysis, lethargy, hunched posture), using carbon dioxide asphyxiation followed by cervical dislocation.
Wild animals	This study did not involve wild animals
Field-collected samples	This study did not involve field-collected samples
Ethics oversight	Animal studies were approved by the Institutional Animal Care and Use Committee at Washington University School of Medicine

Note that full information on the approval of the study protocol must also be provided in the manuscript.

Flow Cytometry

Plots

Confirm that:

☒ The axis labels state the marker and fluorochrome used (e.g. CD4-FITC).

☒ The axis scales are clearly visible. Include numbers along axes only for bottom left plot of group (a 'group' is an analysis of identical markers).

☒ All plots are contour plots with outliers or pseudocolor plots.

☒ A numerical value for number of cells or percentage (with statistics) is provided.

Methodology

Sample preparation	Cell lines and T cells from in vitro experiments were directly transferred into flow cytometry tubes and washed once prior to staining with fluorescently labeled antibodies for 15 minutes at room temperature. For peripheral blood evaluation, 50ul was added to 2ml of RBC lysis buffer in flow cytometry tubes and incubated for 10 minutes at room temperature, followed by a single wash and addition of Fc block (BioLegend #101320), then antibody staining. Spleens were macerated over a 70um cell strainer using the piston from a 3ml syringe to create single cell suspensions, after which 1-2e6 cells were transferred to flow cytometry tubes and washed once prior to Fc block and antibody staining.
Instrument	All samples were run on either an Attune NxT Flow Cytometer (Thermo Fisher Scientific) or a ZE5 (Yeti) cytometer (Bio-Rad).
Software	Data analysis was performed using FlowJo v10.6.1 (Tree Star Inc.) or FCS Express v7.08.0018 (De Novo Software).
Cell population abundance	Post-sort purity of human and mouse CAR T cells were determined by flow cytometry.

Gating strategy

Initial FSC-A/SSC-A gates were drawn on the cell populations, excluding debris, followed by FSC-A/FSC-H gate to define single cells. Dead cells were excluded using live/dead yellow staining. Boundaries between positive and negative cells were established using either the appropriate antigen-negative cell controls (e.g. UTD cells to define CAR+ T cells) or fluorescence-minus-one controls (e.g. for memory T cell markers).

☒ Tick this box to confirm that a figure exemplifying the gating strategy is provided in the Supplementary Information.
